# *Candida auris* Outbreak in a COVID-19 Specialty Care Unit — Florida, July–August 2020

**DOI:** 10.15585/mmwr.mm7002e3

**Published:** 2021-01-15

**Authors:** Christopher Prestel, Erica Anderson, Kaitlin Forsberg, Meghan Lyman, Marie A. de Perio, David Kuhar, Kendra Edwards, Maria Rivera, Alicia Shugart, Maroya Walters, Nychie Q. Dotson

**Affiliations:** ^1^Division of Healthcare Quality Promotion, National Center for Emerging and Zoonotic Infectious Diseases, CDC; ^2^Bureau of Epidemiology, Division of Disease Control and Health Protection, Florida Department of Health; ^3^Division of Foodborne, Waterborne, and Environmental Diseases, National Center for Emerging and Zoonotic Infectious Diseases, CDC; ^4^Office of the Director, National Institute for Occupational Safety and Health, CDC; ^5^Health Systems and Worker Safety Task Force, CDC COVID-19 Response Team; ^6^Bureau of Public Health Laboratories, Division of Disease Control and Health Protection, Florida Department of Health.

*On January 8, 2021, this report was posted online as an *MMWR *Early Release.*

In July 2020, the Florida Department of Health was alerted to three *Candida auris* bloodstream infections and one urinary tract infection in four patients with coronavirus disease 2019 (COVID-19) who received care in the same dedicated COVID-19 unit of an acute care hospital (hospital A). *C. auris* is a multidrug-resistant yeast that can cause invasive infection. Its ability to colonize patients asymptomatically and persist on surfaces has contributed to previous *C. auris* outbreaks in health care settings (*1*–*7*). Since the first *C. auris* case was identified in Florida in 2017, aggressive measures have been implemented to limit spread, including contact tracing and screening upon detection of a new case. Before the COVID-19 pandemic, hospital A conducted admission screening for *C. auris* and admitted colonized patients to a separate dedicated ward.

Hospital A’s COVID-19 unit spanned five wings on four floors, with 12–20 private, intensive care–capable rooms per wing. Only patients with positive test results for SARS-CoV-2, the virus that causes COVID-19, at the time of admission were admitted to this unit. After patient discharge, room turnover procedures included thorough cleaning of all surfaces and floor and ultraviolet disinfection. In response to the four clinical *C. auris* infections, unit-wide point prevalence surveys to identify additional hospitalized patients colonized with *C. auris* were conducted during August 4–18; patients on all four floors were screened sequentially and rescreened only if their initial result was indeterminate. Hospital A’s infection prevention team, the Florida Department of Health, and CDC performed a joint investigation focused on infection prevention and control at hospital A that included observation of health care personnel (HCP) use of personal protective equipment (PPE), contact with and disinfection of shared medical equipment, hand hygiene, and supply storage. This activity was reviewed by CDC and was conducted consistent with applicable federal law and CDC policy.*

Among 67 patients admitted to the COVID-19 unit and screened during point prevalence surveys, 35 (52%) received positive test results. Mean age of colonized patients was 69 years (range = 38–101 years) and 60% were male. Six (17%) colonized patients later had clinical cultures that grew *C. auris*. Among patients screened who had available medical records (20), two (10%) were admitted directly from a long-term care facility and eight (40%) died within 30 days of screening, but whether *C. auris* contributed to death is unknown (Table).

**TABLE:** Demographic and clinical characteristics of patients colonized with *Candida auris* in a COVID-19 specialty care unit identified during screening at an acute care hospital (N = 35) — Florida, August 4–18, 2020

Characteristic (no. with available information)	No. (%)*
**Sex (35)**	
Female	14 (40)
Male	21 (60)
**Mean age, yrs (range) (35)**	69 (38–101)
**Clinical culture with *C. auris* during admission**^†^ **(35)**	6 (17)
**Mortality within 30 days of screening**^§^ **(20)**	8 (40)
**Admitted from long-term care facility (20)**	2 (10)
**Medical devices present at time of screening (20)**	
Central venous catheter	16 (80)
Ventilator	11 (55)
Nasogastric/Gastric tube	11 (55)
Urinary catheter	11 (55)
**Underlying conditions (20)**	
Diabetes	12 (60)
Chronic wound/wound care	4 (20)
Malignancy	3 (15)
Chronic kidney disease	3 (15)
Chronic lung disease	1 (5)
Cardiac disease	1 (5)
No underlying conditions	4 (20)
**Known multidrug-resistant organism before screening (20)**	5 (25)
Vancomycin-resistant Enterococci	3 (15)
Extended-spectrum beta-lactamase–producing Enterobacteriaceae	2 (10)
Methicillin-resistant *Staphylococcus aureus*	2 (10)
Carbapenem-resistant Enterobacteriaceae	0 (—)
*Candida auris*	0 (—)

**Abbreviation:** COVID-19 = coronavirus disease 2019.

* Clinical information available for 20 (57%) of 35 patients. Medical records for other patients were not available. Clinical information on this subset might not be representative of all patients.

^†^ Results of clinical cultures with *Candida auris* finalized after colonization was identified by screening during patients’ current admission.

^§^ Contribution of *C. auris* to mortality is unknown.

HCP in the COVID-19 unit were observed wearing multiple layers of gowns and gloves during care of COVID-19 patients. HCP donned eye protection, an N95 respirator, a cloth isolation gown, gloves, a bouffant cap, and shoe covers on entry to the COVID-19 unit; these were worn during the entire shift. A second, disposable isolation gown and pair of gloves were donned before entering individual patient rooms, then doffed and discarded upon exit. Alcohol-based hand sanitizer was used on gloved hands after doffing outer gloves. HCP removed all PPE and performed hand hygiene before exiting the unit.

Investigators observed multiple opportunities for contamination of the base layer of gown and gloves during doffing and through direct contact with the patient care environment or potentially contaminated surfaces such as mobile computers. Mobile computers and medical equipment were not always disinfected between uses, medical supplies (e.g., oxygen tubing and gauze) were stored in open bins in hallways and accessed by HCP wearing the base PPE layer, and missed opportunities for performing hand hygiene were observed.

A combination of factors that included HCP using multiple gown and glove layers in the COVID-19 unit, extended use of the underlayer of PPE, lapses in cleaning and disinfection of shared medical equipment, and lapses in adherence to hand hygiene likely contributed to widespread *C. auris* transmission. After hospital A removed supplies from hallways, enhanced cleaning and disinfection practices, and ceased base PPE layer practices, no further *C. auris* transmission was detected on subsequent surveys.

The COVID-19 pandemic has prompted facilities to implement PPE conservation strategies during anticipated or existing shortages and to use PPE in ways that are not routine (e.g., extended wear and reuse) (*8*). Some health care facilities not experiencing shortages allow extra PPE layers because of the perception of increased protection for HCP. CDC does not recommend the use of more than one isolation gown or pair of gloves at a time when providing care to patients with suspected or confirmed SARS-CoV-2 infection (*9*,*10*). Such practices among HCP might be motivated by fear of becoming infected with SARS-CoV-2 but instead might increase risks for self-contamination when doffing and for transmission of other pathogens among patients and exacerbate PPE supply shortages. When managing SARS-CoV-2 patients in a dedicated ward, HCP should maintain standard practices (e.g., hand hygiene at indicated times and recommended cleaning and disinfection) intended to prevent transmission of other pathogens.^†^^,^^§^ Outbreaks such as that described in this report highlight the importance of adhering to recommended infection control and PPE practices and continuing surveillance for novel pathogens like *C. auris*.

## Footnotes

* 45 C.F.R. part 46.102(l)(2), 21 C.F.R. part 56; 42 U.S.C. Sect. 241(d); 5 U.S.C. Sect. 552a; 44 U.S.C. Sect. 3501 et seq.

† https://www.cdc.gov/infectioncontrol/guidelines/index.html.

§ https://www.cdc.gov/coronavirus/2019-ncov/hcp/infection-control.html.
